# Phytochemical Constituents and Allelopathic Potential of *Parthenium hysterophorus* L. in Comparison to Commercial Herbicides to Control Weeds

**DOI:** 10.3390/plants10071445

**Published:** 2021-07-15

**Authors:** Mst. Motmainna, Abdul Shukor Juraimi, Md. Kamal Uddin, Norhayu Binti Asib, A. K. M. Mominul Islam, Muhammad Saiful Ahmad-Hamdani, Mahmudul Hasan

**Affiliations:** 1 Department of Crop Science, Faculty of Agriculture, Universiti Putra Malaysia, Serdang 43400, Selangor, Malaysia; gs51794@student.upm.edu.my (M.M.); s_ahmad@upm.edu.my (M.S.A.-H.); gs53801@student.upm.edu.my (M.H.); 2 Department of Land Management, Faculty of Agriculture, Universiti Putra Malaysia, Serdang 43400, Selangor, Malaysia; mkuddin@upm.edu.my; 3 Department of Plant Protection, Faculty of Agriculture, Universiti Putra Malaysia, Serdang 43400, Selangor, Malaysia; norhayuasib@upm.edu.my; 4 Department of Agronomy, Faculty of Agriculture, Bangladesh Agricultural University, Mymensingh 2202, Bangladesh; akmmominulislam@bau.edu.bd

**Keywords:** allelopathy, phytochemicals, *P. hysterophorus*, germination, growth

## Abstract

The allelopathic effect of various concentrations (0, 6.25, 12.5, 50 and 100 g L^−1^) of *Parthenium hysterophorus* methanol extract on *Cyperus iria* was investigated under laboratory and glasshouse conditions. No seed germination was recorded in the laboratory when *P. hysterophorus* extract was applied at 50 g L^−1^. In the glasshouse, *C. iria* was mostly injured by *P. hysterophorus* extract at 100 g L^−1^. The phytochemical constituents of the methanol extract of *P. hysterophorus* were analyzed by LC-ESI-QTOF-MS=MS. The results indicated the presence of phenolic compounds, terpenoids, alkaloids, amino acids, fatty acids, piperazines, benzofuran, indole, amines, azoles, sulfonic acid and other unknown compounds in *P. hysterophorus* methanol extract. A comparative study was also conducted between *P. hysterophorus* extract (20, 40 and 80 g L^−1^) with a synthetic herbicide (glyphosate and glufosinate ammonium at 2 L ha^−1^) as a positive control and no treatment (negative control) on *Ageratum* *conyzoides*, *Oryza* *sativa* and *C. iria.* The growth and biomass of test weeds were remarkably inhibited by *P. hysterophorus* extract. Nevertheless, no significant difference was obtained when *P. hysterophorus* extract (80 g L^−1^) and synthetic herbicides (glyphosate and glufosinate ammonium) were applied on *A.* *conyzoides.*

## 1. Introduction

*Cyperus iria* L. (family: Cyperaceae) is a smooth, tufted sedge weed of lowland rice worldwide and is also a common weed in upland fields of 22 countries [1]. This weed is also reported to appear in dry, direct-seeded rice fields in 21 countries and wet-seeded rice in 11 countries [2]. The roots of *C. iria* are numerous, yellowish-red, short and fibrous. The leaves are usually shorter than culm, 1–8 mm wide and the inflorescence is simple or compound. A prolific nature (5000 seeds from a single plant) and a very short life cycle of *C. iria* help it to establish a second generation in the same growing season [3,4]. It is estimated that approximately 64% of rice yield reduction occurs due to this weed [5].

Weed management in the crop field is a challenging task in agriculture. Chemical herbicides are mainly preferred by the farmers to control weeds due to their higher efficacy, affordable cost and more rapid out return. The migration of labor away from agriculture to industries or other countries for employment is also a major concern for dependence in some countries [6]. However, the excessive use of synthetic herbicides can lead to an increase in the number of herbicide-resistant biotypes [7], low agricultural production, environmental pollution and health hazards [8,9]. On the other hand, the introduction of allelopathic plants or bio-herbicide develop from allelochemicals can play an important role as a substitute for the chemical dependence on synthetic chemical herbicides to control weeds in sustainable agriculture [10].

Invasive weed species have the potential to release allelopathic substances to the surrounding environments to suppress their neighboring competing plants [11,12,13,14,15]. *Parthenium hysterophorus* L. has taken the shape of a noxious weed and is becoming a threat to crop production, animal husbandry and human health due to its strong allelopathic effects [16,17,18,19]. The isolation and identification of the allelopathic substances from *P. hysterophorus* could be used as a tool for the development of a natural-product-based herbicide for weed control.

Bioassays are generally designed to test the allelopathic properties of a plant species. However, a plant that shows strong phytotoxicity on the target plant species in laboratory conditions might not be so strong in the field condition due to the influence of several environmental factors [20,21]. In this context, two experiments were conducted in both laboratory and glasshouse conditions to evaluate the allelopathic properties of *P. hysterophorus* with a view to developing natural-product-based bioherbicides. The identification of its phytochemical constituents was analyzed by using LC-ESI-QTOF-MS=MS.

## 2. Results

### 2.1. Laboratory Experiment

#### Effect of Methanol Extracts on Germination and Initial Growth of *C. iria*

The results showed that *P. hysterophorus* extracts significantly (*p* ≤ 0.05) reduced the germination percentage as well as coleoptile and radicle length of *C. iria* (Table 1). The inhibitory activity was concentration-dependent. By the application of methanol extracts, the seed germination was significantly (*p* ≤ 0.05) reduced. No seed germination was recorded when *P. hysterophorus* extract was applied at 50 g L^−1^. 

*Parthenium hysterophorus* extract decreased the coleoptile and radicle elongation of *C. iria*. The magnitude of inhibition increased with an increase in extract concentration. At a concentration of 50 g L^−1^ or above, *P. hysterophorus* extract reduced the coleoptile and radicle length of *C. iria* by 100%.

### 2.2. Glasshouse Experiment

#### 2.2.1. Effect of Methanol Extract on Plant Height, Leaf Area and Dry Weight of *C. iria*

Table 2 showed the effect of *P. hysterophorus* methanol extract on the plant height, leaf area and dry weight of tested weeds. Dose-dependent inhibitory activity was also observed here. *Parthenium hysterophorus* showed significant inhibition on plant height at the highest concentration (100 g L^−1^). At the concentration of 100 g L^−1^, *P. hysterophorus* extract 44.40% inhibition was observed on the plant height of *C. iria*. A decline in leaf area of the tested weed was also observed with an increase in *P. hysterophorus* methanol extract concentration. The leaf area inhibition of *C. iria* ranged from 7.63 to 52.03% from 6.25 g L^−1^ to 100 g L^−1^ concentrations of *P. hysterophorus* extract. The control obtained the highest dry weight. The extract reduced 60.81% of the dry weight of *C. iria* at 100 g L^−1^ compared to the control.

#### 2.2.2. Effect of Methanol Extract on Fv/Fm, Photosynthesis Rate, Stomatal Conductance and Transpiration Rate of *C. iria*

No significant difference was observed when *C. iria* was treated with 6.25 and 12.5 g L^−1^ of *P. hysterophorus* extract (Table 3). The extract reduced the Fv/Fm value by 46.32% at 100 g L^−1^. The significant effect of extracts concentrations was observed on the photosynthesis, stomatal conductance and transpiration rate of *C. iria*. The photosynthesis rate of *C. iria* was inhibited by 44.41% when treated with the highest concentrations (100 g L^−1^) of *P. hysterophorus* extract. The lowest stomatal conductance (0.25 mol m^−2^ s^−1^) was recorded at 100 g L^−1^, and the inhibition value was 39.63% (Table 4). The lowest transpiration rate was observed at the highest concentration (100 g L^−1^), and the inhibition value was 40.98%. 

### 2.3. Identification of Phytotoxic Components from Methanol Extract of P. hysterophorus

**Figure 1:**
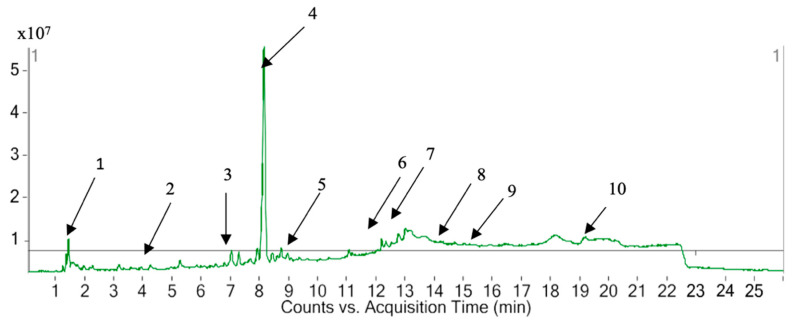
LC-MS chromatograms chemical compounds of *P. hysterophorus* in the positive ion mode (1. Valine, 2. umbelliferone, 3. parthenin, 4. 9-hydroxyellipticine, 5. laciniatin, 6. phytosphingosine, 7. tridecylglycerol, 8. phthalic anhydride, 9. eicosasphinganine, 10. N,N-bis (2-hydroxyethyl) stearylamine).

**Figure 2:**
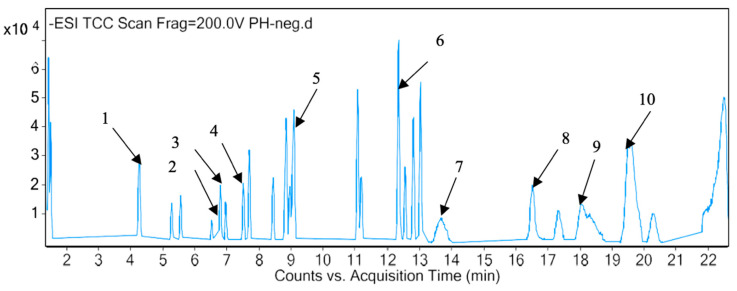
LC-MS chromatograms chemical compounds of *P. hysterophorus* in the negative ion mode (1. Quinic acid, 2. hymonoxynin, 3. chlorogenic acid, 4. isochlorogenic acid, 5. laciniatin, 6. Rishitin, 7. 4-dodecylbenzenesulfonic acid, 8. lauryl sulfate, 9. tridecyl-benzenesulfonic acid, 10. 4-undecyl benzene sulfonic acid).

LC-MS analyses of *P. hysterophorus* methanol extract revealed the presence of 82 known compounds that appeared between 1 and 20 mins. The list of proposed compounds with their retention times, molecular formula, polarity and mass fragment (m/z) is shown in Table 4. For most of the constituents, [M-H]^+^ and [M-H]^−^ ions were observed. The total ion current chromatography in positive and negative ESI mode is shown in Figure 1 and Figure 2. Eight amino acids (Valine, Lajollamide A, Alaptide, Arginyl-tyrosyl-aspartic acid, Thyroliberin N-ethylamide, Hexadecasphinganine, Phytosphingosine and Eicosasphinganine) were identified, which usually provides [M-H]^+^ ions as the best peak positive ESI mode. The amino acids were identified at 1.436, 7.641, 8.004, 8.435, 11.844, 11.996, 12.034, 15.406 min, with 117.0802, 565.4206, 182.1063, 452.2022, 390.2011, 273.2672, 317.2935, 329.3298 m/z, respectively, in the positive ionization mode. A total of seven phenolic compounds (Umbelliferone, Quinic Acid, Chlorogenic acid, Oleacein, Isochlorogenic acid A, Laciniatinand Phthalic anhydride) and three terpenoids (Parthenin, Dehydroleucodine and Rishitin) were also identified. Among the phenolic compounds, chlorogenic acid (C_16_H_18_O_9_) was detected with its [M-H]^−^ ion at 6.939 min with 354.0957 m/z. In positive ionization mode, parthenin (C_15_H_18_O_4_) was detected at 7.006 min with 262.1202 m/z. A fragment ion at 262.1122 m/z was displayed for 9-hydroxyellipticine (alkaloid) in positive ionization mode at 8.136 min. A number of other organic compounds were also detected in *P. hysterophorus* (Table 4). Descyclopropyl Abacavir (C_11_H_14_N_6_O) is a carbohydrate and was detected from the extract at 8.055 min 246.1225 m/z. At 229.24 m/z, Lauramine oxide (C_14_H_31_NO) was identified as a detergent at 12.193 min. Glycolipid (Glyceryl sulfoquinovoside, C_9_H_18_O_10_S) and glycoside (Dihydrophaseic acid 4-O-beta-D-glucoside, C_21_H_32_O_10_) were identified at 1.418 and 5.253 min with 318.063 and 444.1998 m/z, respectively in the negative ionization mode. One ketone (Angoletin, C_18_H_20_O_4_) was also identified in the positive ionization mode at 14.691 with 300.1357 m/z. Two sulfonic acids, namely, 4-dodecylbenzenesulfonic acid (C_18_H_30_O_3_S) and Benzenesulfonic acid, tridecyl- (C_19_H_32_O_3_S) at 13.633 and 18.267 min with 326.1916 and 312.2282 m/z in negative and positive ionization modes, respectively.

### 2.4. Efficacy of P. hysterophorus Extract in Comparison with Commercial Herbicides

**Figure 3:**
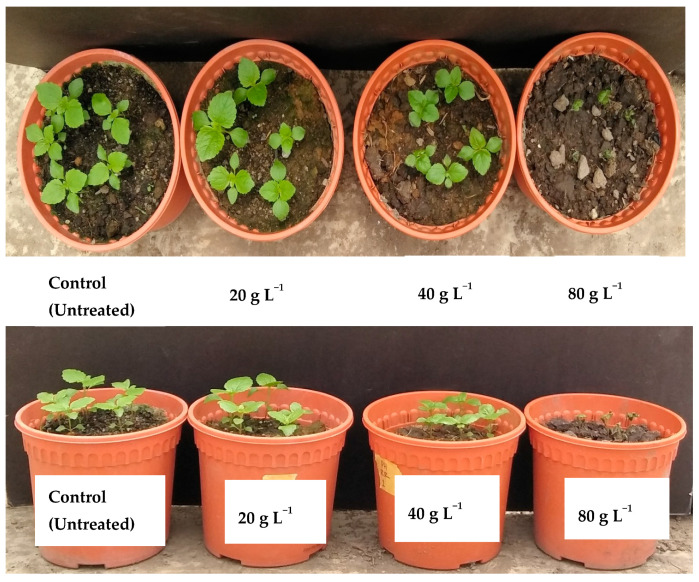
Effect of *P. hysterophorus* extract on *A. conyzoides* at 24 h after spray.

**Figure 4:**
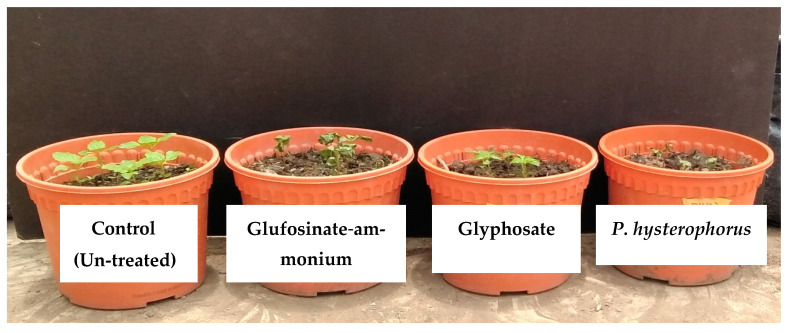
Effect of *P. hysterophorus* extract at 80 g L^−1^ concentration on *A. conyzoides* at 24 h after spray compared with glufosinate-ammonium and glyphosate herbicides.

All treatments had significant effects (*p* ≤ 0.05) on plant height and fresh and dry weight (Table 5). The phytotoxicity effects of *P. hysterophorus* and synthetic herbicide on *A. conyzoides*, *C. iria* and *O. sativa* were evaluated based on visual observation at 21 days after spray (Table 5). The visual injury of *A. conyzoides* was higher compared to *C. iria* and *O. sativa* at the applied concentrations of *P. hysterophorus* methanol extract. At the highest concentration (80 g L^−1^), *A. conyzoides*, *C. iria* and *O. sativa* were injured severely with an injury rating scale of 9.00, 5.25 and 4.50, respectively. *Cyperus iria* and *O. sativa* were alive and showed either green foliage or minor chlorosis or minor leaf curling at the lowest concentration (20 g L^−1^). All tested weeds died after treated with synthetic herbicide (glyphosate and glufosinate ammonium). However, only *A.* *conyzoides* died when *P. hysterophorus* was sprayed at 80 g L^−1^ (Figure 3 and Figure 4).

The plant height of *A.* *conyzoides*, *C. iria* and *O. sativa* was inhibited by 54.32%, 37.71% and 26.08%, respectively, when treated with *P. hysterophorus* extract at 40 g L^−1^. The complete inhibition of plant height of *A.* *conyzoides* was observed on those pots where 80 g L^−1^ of *P. hysterophorus* extract was sprayed, whereas 42.97% and 41.02% plant height inhibitions were observed for *C. iria* and *O. sativa*, respectively, at the same concentration. In general, there was a reduction in the fresh and dry weights of treated weeds in pots receiving *P. hysterophorus* extract. The differences in inhibitory activity among the three doses, *viz.* 20, 40 and 80 g L^−1^ of *P. hysterophorus,* on the fresh and dry weight of weeds, were significant. The dry weights of *A.* *conyzoides*, *C. iria* and *O. sativa* were inhibited by 98.63%, 63.80% and 62.76%, respectively, when *P. hysterophorus* extract was sprayed at 80 g L^−1^. This result exhibited that there is no significant difference between the foliar spray of *P. hysterophorus* at 80 g L^−1^ and positive control when applied on *A.* *conyzoides*, whereas *C. iria* and *O. sativa* were less sensitive to *P. hysterophorus* extract compared to the positive control.

## 3. Discussion

The allelopathic potential of *P. hysterophorus* on *C. iria* was studied in this study. The methanol extract of *P. hysterophorus* influenced *C. iria* seedling growth and germination percentages. The extracts had a dose-dependent effect on the germination percentage, coleoptile and radicle growth of the tested weed. Plant extracts are hypothesized to impede the germination process due to the osmotic effects on the fate of imbibition, which in turn reduce the commencement of germination and, in particular, cell elongation [22]. *C. iria* seed germination and seedling growth were completely suppressed by 50 g L^−1^ of *P. hysterophorus* extract. Batish et al. [23], Singh et al. [24] and Mersie and Singh [25] all observed that *P. hysterophorus* extract or its residues inhibited the growth and development of several field crops. Furthermore, when compared to germination percentage and the coleoptile length, the radicle length of the test species was more sensitive to extracts. As radicles are the first organ to be exposed to phytochemicals and have more permeable tissue than other organs [21,26,27], and/or low mitotic division in the root apical meristem [28], radicle growth is more sensitive to allelopathic plant extract. Furthermore, phytochemicals can inhibit the development of radicle tissues and endoderm by affecting genes involved in cellular characterization [29].

The glasshouse experiment gave more support for the high allelopathic potential of *P. hysterophorus* extract seen in the lab. The results revealed that extracts of *P. hysterophorus* at 50 and 100 g L^−1^ greatly showed the growth of 21-day-old *C. iria*. At the mature stage of *C. iria*, the maximum concentration (100 g L^−1^) of *P. hysterophorus* extract resulted in the greatest decrease. Many researchers from all around the world have demonstrated dose-dependent inhibitory activity [21,27,30,31]. Only untreated *C. iria* continued flowering 21 days after spray, indicating that allelochemicals stress may have suppressed the other treated plants. Aslam et al. [32] investigated the phytotoxic effect of *Calatropis procera, Peganum harmala* and *Tamarix aphylla* on mustard and wheat shoot and root length, finding that wheat was susceptible to all three extracts at all dosages.

As the concentration of *P. hysterophorus* extract was raised, reduced dry weights and leaf area were reduced. The reduction in plant height and leaf area was discovered to be associated with a reduction in total dry weight. Several studies show that different extracts reduce the leaf area of plant species [33,34]. The dry weight of soybeans was greatly changed by the castor beans leaf aqueous extract, according to Da Silva et al. [35].

Foliar spray of *P. hysterophorus* extract reduced the Fv/Fm, photosynthesis rate, stomatal conductance and transpiration of *C. iria*. The value of Fv/Fm was significantly decreased by the foliar spray of *P. hysterophorus* extract. Thylakoid membrane damage and inhibition of energy transfer from antenna molecules to reaction centers can lead to photo-inhibition damage and lower Fv/Fm [36]. Allelochemicals can significantly affect the performance of thylakoid electron transport during light reactions, stomatal control of carbon dioxide and the carbon cycle in dark reactions [37].

The reduction in leaf photosynthesis was attributed to a decrease in photosynthetic metabolites, carboxylation efficiency, impairment of chloroplast activity, increase in enzyme activities [38] and production of ROS caused impediment of photosynthetic mechanism [39]. Stomatal control is a vital property through which the plants limit water loss and gas exchange. These features are influenced by several determinants, including stress [40], and indicate the lower photosynthetic efficiency of plants. The carboxylation and water-use efficiency was also reduced in the plants subjected to *P*. *hysterophorus* extract.

The reduction in the transpiration rate is certainly associated with stomatal conductance. This study reveals that *P*. *hysterophorus* extract played a notable role in decreasing the transpiration rate for test plants at different exposure times. The concentration of phenolic acids resulted in a decline in overall water utilization and transpiration of cucumber seedlings in a linear manner [41]. The solution of cinnamic acid and benzoic acids decreased the stomatal conductance and transpiration of cucumber seedlings [42].

It was also observed in the present study that the application of plant extracts in laboratory conditions caused more inhibition compared to glasshouse as a foliar spray. Al-Humaid and El-Mergawi [43] also reported the same. The inhibition by foliar spray may occur through various mechanisms, such as a decreased rate of ion absorption, hormone and enzyme activity, cell membrane permeability and certain physiological processes, e.g., photosynthesis, respiration and protein formation [44]. Thus, the seedling and mature stage of target plants may vary in their sensitivities to plant extracts.

In this research, the methanol extract of *P. hysterophorus* was also investigated for the identification of active phytochemical constituents using LC-MS QTOF and also for their allelopathic potentiality on *C. iria*. Methanol was reported to be an efficient extraction solvent of lower molecular weight polyphenols [45] and a highly efficient solvent for extracting phenolic compounds compared to ethanol [46]. The results indicated the presence of phenolic compounds (flavonoids, phenols, coumarins, carboxylic acids, benzoic acids), terpenoids, alkaloids, amino acids, fatty acids, piperazines, benzofuran, indole, amines, azoles, sulfonic acid and other unknown compounds in *P. hysterophorus*. Among the proposed compounds, some of them have been reported as toxins in different studies. The hydroxyl group of phenolic compounds is directly attached to an aromatic ring. Phenolic allelochemicals are major allelochemicals that inhibited photosynthesis in plants [42] and modified the permeability of root cell membranes, decreased energy metabolism and inhibited cell division and root branching [47]. Research studies revealed that phenolic compounds from *Chenopodium murale* L. affect the growth and macromolecule content in chickpeas and peas [48].

Umbelliferone, a coumarin derivative, was found in *P. hysterophorus,* and, as Pan et al. [49] reported, it shows strong inhibition on lettuce and two field weeds, *Setaria viridis* and *Amaranthus retroflexus*. Phthalic anhydride, another compound of *P. hysterophorus,* formed Phthalic acid in the presence of water, which inhibited the fruit germination of *Lactuca sativa* L. [50]. Three terpenoids (Parthenin, Dehydroleucodine, Rishitin) and one alkaloid (9-hydroxyellipticine) were also found in *P. hysterophorus* extract. Many past and recent research reports revealed that terpenoids and alkaloids are also known for their allelopathic effect. Parthenin reduced the germination and growth of *Avena fatua* L. and *Bidens pilosa* L. and a dose–response relationship was observed by Batish et al. [51]. Valine is an amino acid found in *P. hysterophorus,* which significantly inhibited peach seedling growth [52]. Some fatty acids, amines and sulfonic acids were also observed in the LC-MS analysis of *P. hysterophorus*.

The efficacy of *P. hysterophorus* extract was increased with an increasing application rate. Similarly, the extract phytotoxicity level of *Zingiber officinale* increased with increasing concentration [53]. At 80 g L^−1^*, P. hysterophorus* extract produced similar efficacy to glyphosate and glufosinate on *A. conyzoides*. Many researchers found the efficacy of bioherbicide for weed control. For instance, *Aglaia odorata* leaf extract has bioherbicide properties that can hinder the growth and development of weeds [54].

Furthermore, the results also indicated that the inhibition magnitude of applied methanol extract of *P. hysterophorus* was species-dependent. The selectivity of an herbicide depends on application rate, the growth stage and morphological characteristics of the target plants and other environmental factors, which might affect the absorption, translocation and metabolism of the herbicide [55].

## 4. Materials and Methods

**Figure 5:**
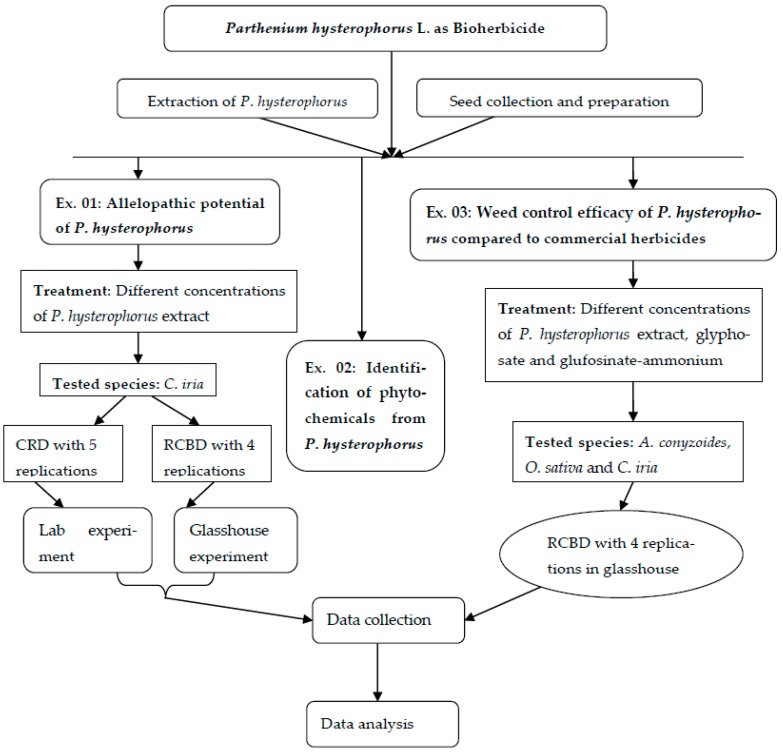
Graphical scheme of study design.

Graphical scheme of experimental design was presented in Figure 5.

### 4.1. Test Plants

*Cyperus iria* L. (Rice flatsedge) *(voucher specimen#UPMWS019)*, *Ageratum conyzoides* L. (Billygoat-weed) *(voucher specimen#UPMWS001),* Oryza sativa f. spontanea Roshev (Weedy rice) *(voucher specimen#UPMWS025)* were collected from the rice field of Sekinchan, Kuala Selangor, Selangor, Malaysia.

### 4.2. Extraction Procedure

The extraction was carried out conducted at Universiti Putra Malaysia’s Weed Science Laboratory, which is a part of the Department of Crop Science. Methanol extracts were prepared using the method reported by Aslani et al. [56]. *Parthenium hysterophorus* (*voucher specimen#UPMWS0031*) was obtained at its matured stage in Ladang Infoternak, Sungai Siput, Perak, Malaysia. The plants were properly washed under running tap water to remove dust particles and other debris, and then air-dried for 3 weeks in open trays under shaded conditions at room temperature (25 ± 1 °C). In a Willey mill, the plants were then chopped and crashed. An amount of 100 g powder of *P*. *hysterophorus* was soaked in a conical flask with 1000 mL methanol: distilled water (80:20, *v*/*v*%) and the flask was wrapped in paraffin. An Orbital shaker was used to shake the flask for 48 h at room temperature (25 ± 1 °C). The solution was filtered through four layers of cheesecloth before being centrifuged at 3000 rpm for 1 hour. Then, a 0.2 mm Nalgene filter was used (Becton Dickinson Labware, Lincoln Park, NJ) to re-filter the solution. A rotary evaporator was used at 40 °C to evaporate the methanol from the extract. The mean extraction yield was 18.56 g from 100 g powdered sample of *P*. *hysterophorus.*

Extraction percentage = [Extract weight (g)/powder weight (g)] × 100(1)

The crude sample (20 mg) was diluted into 100% HPLC GRADE methanol (20 mL) and filtered with 0.2-μm, 15-mm syringe filters (Phenex, Non-sterile, Luer/Slip, LT Resources, Malaysia) for LC-QTOF-MS/MS analysis.

### 4.3. Laboratory Bioassay

From January to March 2019, the experiment was carried out in a growth chamber at the Seed Technology Laboratory, Department of Crop Science, Universiti Putra Malaysia (3°02′ N, 101°42′ E, 31 m elevation). Seeds were gathered that were healthy and uniform, then soaked for 24 h in 0.2 percent potassium nitrate (KNO_3_), rinsed with distilled water and incubated at room (24–26 °C) temperature until the radicle emerged for about 1 mm. Twenty uniform pre-germinated *C. iria* seeds were inserted in disposable plastic Petri dishes with a 9.0-cm-diameter and two sheets of Whatman No. 1 filter paper. After that, the filter paper on the Petri dishes was wetted and soaked with 10 mL of *P. hysterophorus* methanol extracts at six different concentrations: 0 (distilled water only), 6.25, 12.5, 25, 50 and 100 g L^−1^. The treatment was replicated 5 times in a completely randomized design. The Petri dishes were then incubated under fluorescent light (8500 lux) in a growth chamber at 30/20 °C (day/night) with a 12 h/12 h (day/night cycle). The relative humidity ranged from 30% to 50%. To facilitate gas exchange and avoid anaerobic conditions, the lids of the Petri dishes were not sealed.

All seedlings germination %, coleoptile and radicle length were assessed after 7 days. Image J software [57] was used to measure the length of the coleoptile and radicle, and the inhibitory effect was calculated using the equation below [56]:

I = 100 (C−A)/C(2)

where “I” represents the percent inhibition, “C” represents the mean length of coleoptile and radicle of the control and “A” is the mean length of coleoptile and radicle of the methanol extracts treated seeds.

### 4.4. Glasshouse Experiment

The glasshouse experiment took place at Universiti Putra Malaysia’s Faculty of Agriculture in Ladang 15 from April to June 2020. The effects of foliar application of *P. hysterophorus* methanol extracts on the growth and development of *C. iria* were investigated. Pre-germinated seeds were placed in each pot (15 cm diameter × 12 cm height) and covered with 1 cm soil, then moistened with water. Only five healthy seedlings of equal size were maintained in each pot after germination. With four replications, the pots were arranged in a randomized complete block design. Methanol extracts of *P. hysterophorus* were sprayed on examined plants (2–3 leaf stage) at doses of 6.25, 12.5, 25, 50 and 100 g L^−1^ concentrations on tested plants (2–3 leaf stage) using a 1 L multipurpose sprayer (Deluxe pressure sprayer). Water was used to make spray volume (100 mL m^−2^) [22]. At two-day intervals or when the soil became dry, plants in the control treatment were sprayed with 200 mL water without extract. Three weeks after spray, plant height, leaf area, dry weight, Fv/Fm, photosynthesis rate, transpiration and stomatal conductance were determined. Plant height was measured using 1 m ruler from the ground level in the pot. The leaf area was determined using leaf area meter (LI-3000, Li-COR, USA) and expressed as cm^2^ plant^−1^. Samples were dried in an oven at 60 °C for 72 h; then, dry weights were determined using a digital balance. The efficiency of photosystem II in each leaf was measured with a Multi-Function Plant Efficiency Analyser (Hansatech Instruments, King’s Lynn, United Kingdom). The Fv parameter (variable fluorescence) was calculated as the difference between the Fm (maximum fluorescence) and Fo (minimum fluorescence). The rate of photosynthesis, transpiration and stomatal conductance were measured from randomly selected four leaves from each test weed species using LICOR (LI-6400XT) portable photosynthesis system, (LI-COR-Inc Lincoln, Nebraska, USA) between 9:00 am to 11:00 am under bright daylight. The measurements were taken on the abaxial surface at CO_2_ flow rate of 400 μmol m^−2^ s^−1^ and the saturating photosynthetic photon flux density (PPFD) was 1000 mmol m^−2^ s^−1^ [58].

Another experiment was conducted to compare the phytotoxicity level of *P. hysterophorus* with synthetic herbicides. Therefore, the seeds of *A.* *conyzoides*, *C. iria* and *O. sativa* were seeded in the pots (15 cm diameter) and moistened with tap water. After germination, five equal-sized healthy seedlings were kept in each pot. The pots were arranged in a randomized complete block design with four replications. Methanol extracts of *P. hysterophorus* were sprayed with 20, 40 and 80 g L^−1^ concentration on tested plants (4–6 leaf stage for broadleaf and 2–3 for grasses and sedges). Plants in the negative control treatment were sprayed with 200 mL water without extract at 2 day intervals or when the soil became dry. Plants in the positive control treatment were sprayed with glyphosate 41% a.i. *(*Roundup*^®^)* and glufosinate-ammonium 13.5% a.i. (Basta^®^) without extract (2 L ha^−1^/4.4 mL L^−1^) at the same time when *P. hysterophorus* was sprayed.

Injury symptoms, plant height (cm) and fresh and dry weights (g pot^−1^) were measured 3 weeks after spray. Injury symptoms were visually evaluated on test weeds using the European Weed Control and Crop Injury Evaluation scale (Table 6).

### 4.5. LC-QTOF-MS/MS Analysis

Agilent 1290 Infinity LC system coupled to Agilent 6520 Accurate-Mass Q-TOF mass spectrometer with dual ESI source was used for analyzing chemical constituents from the methanol extract of *P. hysterophorus*. The types of the column, solvent systems and MS parameters were optimized for better analysis of the chemical profiling. ACQUITY UPLC BEH C18 column (150 mm × 2.1 mm × 3.5 μm) was selected and held at 50 °C with a constant flow rate of 0.4 mL min^−1^ for providing fast and efficient separations at lower column pressures [60] and total LC run time was 26 min. Sample elution was performed in a gradient manner using a mobile phase comprised of water (LC-MS Grade) containing 0.1% Formic acid (solvent A) and acetonitrile (LC-MS Grade) containing 0.1% Formic acid (solvent B). Nebulizer pressure was 40 psi, drying gas flow and temperature was set at 10 L min^−1^ and 325 °C, respectively, to perform the MS/MS experiments. In order to obtain the most sensitive ionization effect for analytes, positive and negative ion modes were investigated at different collision energy (CE) to optimize the signals and obtain maximal structure information from the ions for the mass range of 100–3200 m/z. Data processing was performed by Mass Hunter Qualitative Analysis software and peak identification was carried out based on comparison with literature values and online database [61].

### 4.6. Statistical Analysis

For all trials, a one-way analysis of variance (ANOVA) was used to see if there were any significant differences between the treatments and the control. The Tukey test with a 0.05 probability level was used to pool the differences between the treatment means. The analysis was carried out using SAS (Statistical Analysis System) software (version 9.4).

## 5. Conclusions

The current study reveals that the *P. hysterophorus* extract was capable of inhibiting the germination and growth of weeds and also confirmed the herbicidal potential compared with synthetic herbicides. The presence of 82 known compounds was also confirmed in the extract of *P. hysterophorus* and some of them have been reported as toxins in different studies. The great efficacy and selectivity of this weed could be characterized as a natural product to control weeds. The use of plant-based bioherbicide for weed management can increase crop yields as well as provide an alternative method of sustainable weed management. The most phytotoxic compounds from *P. hysterophorus* can be synthesize to develop new natural herbicides with novel modes of action. Metabolomics identification and the isolation of the major potential allelopathins, coupled with formulation techniques via multiple surfactants/nano-formulation, are also required to enhance the penetration and absorption of active compounds.

## Figures and Tables

**Table 1:** Effects of *P. hysterophorus* on germination, coleoptile and radicle length of *C. iria.*

Dose (g L^−1^)	Germination (%)	Coleoptile Length (cm)	Radicle Length (cm)
0.00	100.00a (0)	1.51a (0)	1.66a (0)
6.25	80.00b (20)	1.20b (20.72)	1.10b (33.68)
12.5	47.00c (53)	0.86c (43.14)	0.60c (64.02)
25	19.00d (81)	0.36d (76.24)	0.24d (85.65)
50	0.00e (100)	0.00e (100)	0.00e (100)
100	0.00e (100)	0.00e (100)	0.00e (100)

Data are expressed as means. Means with same letters in the column for concentrations are not significantly different at *p* > 0.05. Values inside the parenthesis are inhibition percentages relative to the control.

**Table 2:** Effect of *P. hysterophorus* methanol extracts on the plant height (cm), leaf area (cm^2^) and dry weight (g pot^−1^) of *C. iria.*

Dose (g L^−1^)	Plant Height	Leaf Area	Dry Weight
0	64.75a (0)	151.05a (0)	5.12a (0)
6.25	63.37ab (2.13)	139.52b (7.63)	4.89ab (4.46)
12.5	62.02ab (4.20)	132.24c (12.44)	4.53b (11.44)
25	57.42b (11.29)	115.22d (23.70)	3.86c (24.55)
50	50.31c (22.31)	91.15e (39.63)	3.00d (41.20)
100	36.00d (44.40)	72.45f (52.03)	2.00e (60.81)

Data are expressed as means. Means with same letters in the column for each extract concentrations are not significantly different at *p* > 0.05. Values inside the parenthesis are inhibition percentages relative to the control.

**Table 3:** Effects of *P. hysterophorus* methanol extract on Fv/Fm, photosynthesis rate (µmol m^−2^ s^−1^), stomatal conductance (mol m^−2^ s^−1^) and transpiration rate (mmol m^−2^ s^−1^) of *C. iria.*

Dose (g L^−1^)	Fv/Fm	Photosynthesis Rate	Stomatal Conductance	Transpiration Rate
0	1.47a (0)	45.14a (0)	0.42a (0)	11.50a (0)
6.25	1.41a (3.90)	43.50ab (3.64)	0.41ab (3.43)	10.83b (5.82)
12.5	1.34a (8.56)	42.50ab (5.86)	0.40ab (6.04)	10.41c (9.52)
25	1.20ab (17.84)	40.00b (11.37)	0.38b (10.07)	9.35d (18.69)
50	1.08ab (26.19)	35.29c (21.86)	0.34c (20.31)	8.20e (28.67)
100	0.79b (46.32)	25.13d (44.41)	0.25d (39.63)	6.79f (40.98)

Data are expressed as means. Means with same letters in the column for each extract concentrations are not significantly different at *p* > 0.05. Values inside the parenthesis are inhibition percentages relative to the control.

**Table 4:** LC-MS profile of methanol extract of *P. hysterophorus*.

Sl. No	RT (min)	Proposed Compound	Molecular Formula	Mass Fragment (m/z)	Polarity
1	1.436	Valine	C_5_H_11_NO_2_	117.0802	Positive
2	1.418	Glyceryl sulfoquinovoside	C_9_H_18_O_10_S	318.063	Negative
3	1.575	Lotaustralin	C_11_H_19_NO_6_	261.1215	Positive
4	3.162	Trazolopride	C_20_H_23_N_5_O_2_	365.1851	Positive
5	3.571	Pirenzepine	C_19_H_21_N_5_O_2_	351.1694	Positive
6	3.92	1-Cyclopropyl-3-[[1-(4-hydroxybutyl)benzimidazol-2-yl]methyl]imidazo [4,5-c]pyridin-2-one	C_21_H_23_N_5_O_2_	377.1848	Positive
7	4.239	Umbelliferone	C_9_H_6_O_3_	162.0317	Positive
8	4.244	Quinic Acid	C_7_H_12_O_6_	192.0638	Negative
9	4.941	Atevirdine	C_21_H_25_N_5_O_2_	379.2002	Positive
10	5.253	Dihydrophaseic acid 4-O-beta-D-glucoside	C_21_H_32_O_10_	444.1998	Negative
11	5.536	2-(2-Ethoxyethoxy)ethanol;4-methylbenzenesulfonic acid	C_13_H_22_O_6_S	306.1136	Negative
12	5.475	4-Azidobenzyl benzyl 1,4-butanediylbiscarbamate	C_20_H_23_N_5_O_4_	397.175	Positive
13	5.823	4-(N-hydroxyamino)-2r-isobutyl-2_S_-(2-Thienylthiomethyl)succinyl-L-Phenylalanine-N-Methylamide	C_20_H_31_NO_3_S_2_	397.176	Positive
14	6.08	Branaplam	C_22_H_27_N_5_O_2_	393.2162	Positive
15	6.257	Pulchellamine G	C_21_H_31_N O_6_	393.2151	Positive
16	6.503	Hymenoxynin	C_21_H_34_O_9_	430.2208	Negative
17	6.939	Chlorogenic acid	C_16_H_18_O_9_	354.0957	Negative
18	7.006	Parthenin	C_15_H_18_O_4_	262.1202	Positive
19	7.006	Gaillardilin	C_17_H_22_O_6_	322.1415	Positive
20	7.006	Dehydroleucodine	C_15_H_16_O_3_	244.1095	Positive
21	7.264	N-Propyl-3-(1,3-thiazol-2-yl)thian-3-amine	C_11_H_18_N_2_S_2_	242.0928	Positive
22	7.266	Oleacein	C_17_H_20_O_6_	320.1252	Positive
23	7.49	Bendazac lysine	C_22_H_28_N_4_O_5_	428.2053	Negative
24	7.641	Lajollamide A	C_30_H_55_N_5_O_5_	565.4206	Positive
25	7.673	Isochlorogenic acid A	C_25_H_24_O_12_	516.127	Negative
26	7.673	Chlorogenic acid	C_16_H_18_O_9_	354.0958	Negative
27	7.897	4-[(6-Chloro-2-naphthalenyl)sulfonyl]-1-[[1-(4-pyridinyl)-4-piperidinyl]methyl]-2 piperazinecarboxylic acid	C_27_H_41_ClN_4_O_6_	552.2699	Positive
28	7.905	N-Chloro-9-(diaminomethylideneamino)-3-hydroxynonanamide	C_10_H_21_ClN_4_O_2_	264.1358	Positive
29	7.908	1-(N-6-Amino-n-hexyl)carbamoylimidazole	C_10_H_19_ClN_4_O	246.1253	Positive
30	8.042	2,4-Toluene Diisocyanate Dimer	C_18_H_12_N_4_O_4_	348.0862	Positive
31	8.044	Alaptide	C_9_H_14_N_2_O_2_	182.1063	Positive
32	8.05	Carbocyclic-3′-amino-ara-adenosine	C_11_H_16_N_6_O_2_	264.1339	Positive
33	8.054	Tris(pyrazolyl)ethane	C_11_H_12_N_6_	228.1118	Positive
34	8.055	Descyclopropyl Abacavir	C_11_H_14_N_6_O	246.1225	Positive
35	8.058	1-Boc-3-oxopiperazine	C_9_H_16_N_2_O_3_	200.1162	Positive
36	8.13	Teroxalene hydrochloride	C_28_H_42_Cl_2_N_2_OS	524.2364	Positive
37	8.132	Ethane;(3-oxo-6′-sulfanylcarbonyloxyspiro [2 -benzofuran-1,9′-xanthene]-3′-yl)oxymethanethioicS-acid;propane	C_31_H_38_O_7_S_2_	586.206	Positive
38	8.133	(2-Aminoethylamino) 2,2-diaminooxyacetate	C_4_H_12_N_4_O_4_	180.0845	Positive
39	8.134	N-[(S)-2-Benzo[1,3]dioxol-5-yl-4-(4-phenyl-piperidin-1-yl)-butyl]-N-methyl-benzenesulfonamide	C_29_H_34_N_2_O_4_S	506.2237	Positive
40	8.135	3-Diazo-1-hexylsulfanyl-1-methylurea	C_8_H_16_N_4_OS	216.1055	Positive
41	8.135	Ethylene oxide-b-maleic hydrazide	C_6_H_12_N_8_O_3_	244.103	Positive
42	8.136	N-[3-(1H-Imidazol-4-yl)propyl]-N′-methylthiourea	C_8_H_14_N_4_S	198.0952	Positive
43	8.136	1-Methylpiperazine-1,4-Diium Bis	C_5_H_14_N_4_O_6_	226.0914	Positive
44	8.136	3-(2-Methylpropylthio)-1H-1,2,4-triazol-5-amine	C_6_ H_12_N_4_S	172.0801	Positive
45	8.136	Benzylamidinoisothiourea	C_9_H_12_N_4_S	208.0792	Positive
46	8.136	1-Amino-3-(propylamino)thiourea	C_4_H_12_N_4_S	148.0798	Positive
47	8.136	9-hydroxyellipticine	C_17_H_14_N_2_O	262.1122	Positive
48	8.136	4-Phenylamino-3-quinolinecarbonitrile deriv. 28	C_27_H_30_Cl_2_N_4_O_4_	544.16	Positive
49	8.136	1-(3-ethyl-1,2,4-thiadiazol-5-yl)azetidin-3-amine	C_7_H_12_N_4_S	184.0793	Positive
50	8.413	1,8,15,22,29,36-Hexaazacyclodotetracontane-2,7,16,21,30,35-hexone	C_36_H_66_N_6_O_6_	678.504	Positive
51	8.415	2,4,6-tris(3-methylbutoxy)-1,3,5-triazine	C_18_H_33_N_3_O_3_	339.2522	Positive
52	8.435	Arginyl-tyrosyl-aspartic acid	C_19_H_28_N_6_O_7_	452.2022	Positive
53	8.636	8-(2,4,6-Trimethoxyphenyl)-9H-purine-2,6-diamine	C_14_H_16_N_6_O_3_	316.1282	Positive
54	8.818	Dimethyl 2-(heptane-1-sulfonyl)butanedioate	C_13_H_24_O_6_S	308.1298	Negative
55	8.721	AC-Ala-gln-ala-pna	C_19_H_26_ N_6_O_7_	450.1864	Positive
56	9.065	Laciniatin	C_17_H_14_O_8_	346.0693	Positive
57	9.067	2-[(3,5-Dinitrobenzoyl)amino]benzoic acid	C_14_H_9_N_3_O_7_	331.0461	Negative
58	9.243	3-Ethyl-1-propyl-8-(1H-pyrazol-4-yl)-1H-purine-2,6(3H,7H)-dione	C_13_H_16_N_6_O_2_	288.134	Positive
59	11.645	Apnea	C_18_H_22_N_6_O_4_	386.1696	Positive
60	11.844	Thyroliberin N-ethylamide	C_18_H_26_N_6_O_4_	390.2011	Positive
61	11.996	Hexadecasphinganine	C_16_H_35_NO_2_	273.2672	Positive
62	12.034	Phytosphingosine	C_18_H_39_NO_3_	317.2935	Positive
63	12.176	Dihydroxyethyllauramine oxide	C_16_H_35_NO_3_	289.262	Positive
64	12.193	Lauramine oxide	C_14_H_31_NO	229.2405	Positive
65	12.308	Rishitin	C_14_H_22_O_2_	222.161	Negative
66	12.316	Dioctylnitrosamine	C_16_H_34_N_2_O	270.2673	Positive
67	12.343	Dodecylacrylamide	C_15_H_29_NO	239.2251	Positive
68	12.349	Tetrabutylurea	C_17_H_36_N_2_O	284.2832	Positive
69	12.703	Aminopregnane	C_21_H_37_N	303.2934	Positive
70	12.778	Tridecylglycerol	C_16_H_34_O_3_	274.2512	Positive
71	13.164	2,3,3-Tris(1,2-diaminoethyl)-2-ethylhexanoic acid	C_14_H_34_N_6_O_2_	318.2769	Positive
72	13.633	4-dodecylbenzenesulfonic acid	C_18_H_30_O_3_S	326.1916	Negative
73	14.691	Angoletin	C_18_H_20_O_4_	300.1357	Positive
74	14.694	Phthalic anhydride	C_8_H_4_O_3_	148.069	Positive
75	15.406	Eicosasphinganine	C_20_H_43_NO_2_	329.3298	Positive
76	16.483	Lauryl sulfate	C_12_H_26_O_4_S	266.1551	Negative
77	16.957	Dodecandial-disemicarbazon	C_14_H_28_N_6_O_2_	312.2282	Positive
78	18.267	Benzenesulfonic acid, tridecyl-	C_19_H_32_O_3_S	340.2072	Negative
79	19.135	3-[5-(3-Dimethylamino-1,2,4-thiadiazol)-yl] quinuclidine	C_11_H_18_N_4_S	238.125	Positive
80	19.496	Benzenesulfonic acid, undecyl-	C_17_H_28_O_3_S	312.176	Negative
81	19.918	N,N-bis(2-hydroxyethyl)stearylamine	C_22_H_47_NO_2_	357.3609	Positive
82	20.245	Benzoyl benzenecarboperoxoate;dodecane-1-thiol;toluene	C_33_H_44_O_4_S	536.2965	Positive

**Table 5:** Effect of *P.* *hysterophorus* on the visual injury, plant height, fresh weight and dry weight of *A.* *conyzoides, C. iria* and *O. sativa.*

	Tested Weeds	*P. hysterophorus*				Synthetic Herbicides	
		0 g L^−1^	20 g L^−1^	40 g L^−1^	80 g L^−1^	Glyphosate	Glufosinate-Ammonium
	* A. * *conyzoides*	1.00d	2.75c	5.50b	9.00a	9.00a	9.00a
Visual injury (Scale)	*C. iria*	1.00e	2.50d	4.00c	5.25b	9.00a	9.00a
	*O. sativa*	1.00e	2.25d	3.00c	4.50b	9.00a	9.00a
	* A. * *conyzoides*	32.00a (0)	24.62b (23.02)	14.62c (54.32)	0.00d (100)	0.00d (100)	0.00d (100)
Plant height (cm)	*C. iria*	64.75a (0)	55.75b (13.58)	44.25c (37.71)	37.00d (42.97)	0.00e (100)	0.00e (100)
	*O. sativa*	67.00a (0)	58.50b (12.68)	49.50c (26.08)	39.53d (41.02)	0.00e (100)	0.00e (100)
	* A. * *conyzoides*	26.45a (0)	18.34b (30.66)	3.14c (88.10)	0.45d (98.28)	0.22d (99.17)	0.27d (98.96)
Fresh weight (g pot^−1^)	*C. iria*	25.95a (0)	20.21b (22.10)	15.70c (39.45)	12.80d (50.60)	0.30e (98.86)	0.50e (98.08)
	*O. sativa*	12.70a (0)	8.89b (29.97)	6.99c (44.93)	5.44d (57.13)	0.14e (98.92)	0.19e (98.48)
	* A. * *conyzoides*	5.13a (0)	3.04b (40.78)	0.50c (90.36)	0.07c (98.63)	0.03c (99.42)	0.05c (99.08)
Dry weight (g pot^−1^)	*C. iria*	6.29a (0)	4.95b (21.12)	3.98c (36.53)	2.28d (63.80)	0.06e (98.97)	0.10e (98.43)
	*O. sativa*	3.36a (0)	2.25b (32.27)	1.75bc (47.49)	1.24c (62.76)	0.03d (99.05)	0.04d (98.77)

Data are expressed as means. Means with same letters in the row are not significantly different at *p* < 0.05. Values inside the parenthesis are inhibition percentages relative to the control.

**Table 6:** Injury rating scale [59].

Scale	Injury (%)	Effects on Weeds
1	0	No effect (all foliage green and alive)
2	1–10	Very light symptoms
3	11–30	Light symptoms
4	31–49	Symptoms not reflected in yield
5	50	Medium
6	51–70	Fairly heavy damage
7	71–90	Heavy damage
8	91–99	Very heavy damage
9	100	Complete kill (dead)

## Data Availability

Not applicable.
